# Cytotoxicity and virulence attributes of *Pseudomonas aeruginosa* isolates from case reports of patients with necrotizing pneumonia

**DOI:** 10.1016/j.rmcr.2025.102329

**Published:** 2025-11-19

**Authors:** T.C. Bolig, S.H. Nozick, C.M.R. Axline, I. Niki, A. Valdes, A. Alisoltanidehkordi, T.L. Turner, F.P.F. Campos, H. Namkoong, P. Riviere, J. Rello, E. Ozer, R. Wunderink, A.R. Hauser

**Affiliations:** a Department of Medicine, Division of Pulmonary and Critical Care, Northwestern University Feinberg School of Medicine, Chicago, IL, USA; b Department of Microbiology-Immunology, Northwestern University Feinberg School of Medicine, Chicago, IL, USA; c Northwestern University, Evanston, IL, USA; d Center for Pathogen Genomics and Microbial Evolution, Robert J. Havey Institute for Global Health, Northwestern University Feinberg School of Medicine, Chicago, IL, USA; e Divisão de Clinica Médica, Hospital Universitário, Universidade de São Paulo, São Paulo, Brazil; f Department of Pulmonary Medicine, Eiju General Hospital, Tokyo, Japan; g Department of Infectious Diseases, Keio University School of Medicine, Tokyo, Japan; h Hôpital Duchenne, Infectious Disease Department, Boulogne-sur-Mer, France; i Centro de Investigación Biomédica en Red de Enfermedades Respiratorias (CIBERES), Instituto Salud Carlos, Madrid, Spain; j Clinical Research in Pneumonia & Sepsis (CRIPS), Vall d'Hebron Institute of Research, Barcelona, Spain; k UR-UM103 IMAGINE, Univ Montpellier, Division of Anesthesia Critical Care Pain and Emergency Medicine, Nimes University Hospital, Montpellier, France; l Department of Medicine, Division of Infectious Diseases, Northwestern University Feinberg School of Medicine, Chicago, IL, USA

**Keywords:** *Pseudomonas aeruginosa,* necrotizing pneumonia, Cytotoxicity, Virulence, Case report, *Galleria*

## Abstract

**Background:**

*Pseudomonas aeruginosa* is a gram-negative bacterium that commonly causes pneumonia, including a rare and aggressive form of lung infection called necrotizing pneumonia (NP). NP is characterized by hemorrhagic necrosis, abscess formation, and cavitation in the lung. The explanation for why some cases of pneumonia progress to NP remains unclear but may involve bacterial or host factors.

**Methods:**

To assess whether some strains of *P. aeruginosa* have unique pathogenic properties that facilitate progression to NP, we obtained six clinical isolates from known cases of *P. aeruginosa* NP. We measured the genetic relatedness of these isolates, their cytotoxicity towards pulmonary epithelial-like cells, and their virulence in *Galleria mellonella*.

**Results:**

The NP isolates did not share a single lineage or sequence type. There was no significant difference in cytotoxicity or *G. mellonella* mortality between NP isolates and a control group of non-NP clinical and reference strains. However, the NP isolates did show a trend towards higher cytotoxicity than the control strains after 3-h of co-incubation with pulmonary epithelial-like cells (16.3 % versus 1.7 %, p = 0.23).

**Conclusions:**

Future studies of NP should focus on larger numbers of patients and bacterial strains to further explore whether NP isolates have enhanced cytotoxicity, examine a broader range of bacterial phenotypes and virulence determinants, and consider the role of host factors.

## 1 Introduction

*Pseudomonas aeruginosa* frequently causes hospital-acquired pneumonia and less commonly community-acquired pneumonia [1]. Rarely, a patient with *P. aeruginosa* pneumonia will progress to a severe manifestation known as necrotizing pneumonia (NP). Pathologically, NP is defined as pneumonia in which hemorrhagic lesions and dead tissue are observed in the lungs [1]. On chest radiographs, pulmonary consolidations that progress to abscesses and cavitations are frequently observed [[2], [3], [4]]. Approximately 1% of pneumonia cases are NP, with higher prevalences noted when imaging studies are carefully examined [5,6]. Men appear to be affected more than women, perhaps because smoking, excessive alcohol use, and liver disease are risk factors for NP [7]. Patients with *P. aeruginosa* NP are at high risk for morbidity and mortality, so early and appropriate antimicrobial therapy is essential. The optimal choice and duration of antibiotic therapy for patients with severe NP pneumonia caused by *P. aeruginosa* remains controversial in the context of multi-drug resistant strains [8].

Perhaps because of its rarity, *P. aeruginosa* NP has not been well studied, and its pathogenesis remains unclear. On one hand, host deficiencies such as immunosuppression or structural lung disease may allow for uncontrolled bacterial multiplication, invasion, and subsequent destruction of pulmonary tissues. Alternatively, rare lineages of *P. aeruginosa* may possess unique pathogenic features or unusual combinations of conventional pathogenic features that cause an exceptional degree of tissue destruction. Such factors may confer the ability to directly lyse host cells, disseminate to deeper tissues, avoid immune clearance, or cause inappropriate vascular clotting. The effector protein ExoU, secreted by the *P. aeruginosa* type III secretion system (T3SS), is one such candidate factor [9]. ExoU is a phospholipase that causes dissolution of host cell membranes, leading to rapid lysis of mammalian cells *in vitro* [10].

To assess whether *P. aeruginosa* strains causing NP possess unique attributes, we obtained six bacterial isolates cultured from patients with these infections. We performed whole-genome sequencing on these isolates and assessed their phylogenetic relationships, measured their abilities to lyse pulmonary epithelial-like cells, and quantified their virulence in a *Galleria mellonella* model system.

## 2 Patients and methods

### 2.1 Patients and *P. aeruginosa* isolates

To identify published cases of *P. aeruginosa* necrotizing pneumonia, a Pubmed search was performed using search terms “*Pseudomonas aeruginosa”* AND “pneumonia” AND (“necrotizing” OR “cavitary”) in January 2017 and periodically thereafter. Articles found in this way were reviewed and used to identify other relevant case reports. Corresponding authors from recent appropriate cases were contacted to determine whether *P. aeruginosa* isolates were available and whether the authors were willing to share them with the corresponding author's laboratory. In this way, isolates from four different NP cases were obtained [3,[11], [12], [13]]. An additional two clinical isolates were obtained from patients enrolled in the Successful Clinical Response in Pneumonia Therapy (SCRIPT) repository at Northwestern University in Chicago, U.S.A. (One of these two isolates has been previously reported [14].) Patient demographics, medical histories, radiographic data, and clinical courses were collected from the published reports or, in the case of SCRIPT patients, from the electronic health record. The study protocol for SCRIPT was approved by the Northwestern University Institutional Review Board (STU00204868). *P. aeruginosa* reference strains PAO1 and PA14 were used as controls, as were 35 previously published isolates cultured from patients in Spain with non-necrotizing pneumonia (designated “PSE” isolates) [[15], [16], [17], [18]].

### 2.2 Genomic analyses

Genomic DNA was harvested from bacteria grown overnight in Lysogenic broth (LB) using Maxwell 16 Cell DNA Purification Kits (Promega, Madison, WI, USA). DNA library preparation was performed using Nextera XT DNA Library Preparation Kits (Illumina, San Diego, CA, USA). Illumina sequencing was performed on a MiSeq or NextSeq-500 instrument. Sequences were deposited in the National Center for Biotechnology Information database (see Supplemental Table 1 for accession numbers). The programs Spine and AGEnt were used to determine the core and accessory genomes [19]. To construct a phylogenetic tree based on core genomes, Illumina reads from each isolate were aligned to the genome sequence of strain PAO1 (NCBI accession GCF_000006765.1) using bwa v0.7.15 [20]. Single nucleotide variants (SNVs) were identified using bcftools v1.9 with a haploid model and skipping bases with quality lower than 25 or alignment quality less than 30. SNVs were further filtered as previously described [21] using the bcftools_filter software (https://github.com/egonozer/bcftools_filter) to remove variants with SNV quality scores less than 200, read consensus less than 75%, read depths less than 5, read numbers in each direction less than 1, or locations within repetitive regions as defined by blast alignment of the reference genome sequence against itself. A maximum likelihood phylogenetic tree was generated from the core genome alignment with IQ-TREE v2.2.0 using the ModelFinder function to estimate the best-fit nucleotide substitution model by means of Bayesian information criterion (BIC) [22,23]. Tree topology was assessed with the ultrafast booststrap (UFboot) method with 1000 replicates [24]. Multilocus sequence typing (MLST) was performed using the PubMLST database (pubmlst.org) for *P. aeruginosa*. To determine the sequence types of the clinical isolates, the genomic sequences were queried against the database for seven housekeeping genes (*acsA*, *aroE*, *guaA*, *mutL*, *nuoD*, *ppsA,* and *trpE*).

### 2.3 Cytotoxicity assays

Cytotoxicity was assessed using quantitative lactate dehydrogenase (LDH) release assays (CyQUANT LDH Cytotoxicity Assay, Invitrogen-ThermoFisher Scientific, Waltham, MA, USA) as previously described [14]. Briefly, approximately 20,000 A549 pulmonary epithelial-like cells were seeded into each well of 96-well tissue culture-treated plates with Dulbecco's modified Eagle medium (DMEM) containing 10% fetal bovine serum. The A549 cells were grown for 22 hours. Then, the DMEM was replaced with RPMI 1640 medium without phenol red. In parallel, *P. aeruginosa* bacteria were inoculated into 5 mL of LB and grown overnight with shaking at 37ᵒC. The bacteria were then pelleted and resuspended in phosphate-buffered saline (PBS) at the appropriate concentration, and 10 μL of bacterial suspension was added to wells to achieve an MOI of ∼10. The signal associated with 100% cell lysis was determined by measuring the LDH released from wells lacking bacteria but treated with 0.05% Triton X-100 detergent. The negative control contained 10 μL of PBS added to the well of A549 cells in place of the bacterial suspension. LDH release was measured at 3 and 8 hours. The percentage of cells lysed was calculated using the following equation: [(LDH activity in sample well)/(LDH activity in Triton X-100-treated wells)] x 100.

### 2.4 Virulence in the *G. mellonella* model

Virulence in *G. mellonella* larvae was measured as previously described [25]. Briefly, bacterial isolates were grown in 5 mL LB overnight at 37ᵒC with shaking. The isolates were then sub-cultured in LB, pelleted, and resuspended in PBS. Estimated doses for injection into *G. mellonella* larvae were determined by diluting bacteria in PBS to an optical density at 600 nm of ∼0.2 (equates to ∼5 × 10^7^ CFU/mL) and making dilutions of PBS to obtain a range of bacterial doses. The bacterial suspensions were injected into the final prolegs of *G. mellonella* larvae. Each inoculum for each strain was injected into 10 larvae. PBS injections served as negative controls. The larvae were placed into petri dishes kept at 37ᵒC and monitored for death hourly. For each dose, the time to reach 50% mortality (LT_50_) was estimated via a custom R-Script. LT_50_ values were then plotted against the natural log of the corresponding doses for each strain. A regression line was fit to these data, and the LT_50_ at a dose of 2000 CFU was estimated from this line, which allowed for virulence comparison between strains.

### 2.5 Statistical analyses

All statistical tests were completed in R (version 4.4.0). Two-tailed *t*-tests were performed to compare means of percent cytotoxicity and LT_50_ values. p values of ≤0.05 were considered significant.

## 3 Results

### 3.1 Summary of cases (Table 1)

Case 1 An 80-year-old man presented to the hospital with a three-day history of severe right-sided chest pain associated with dyspnea and fever. He had recently been diagnosed with autoimmune hemolytic anemia and was taking high-dose prednisone (70 mg daily). *P.* *aeruginosa* grew from his blood culture and his bronchoalveolar lavage (BAL) culture (isolate PA1-Riv). A chest x-ray revealed a right upper lobe cavitary lesion. The patient improved and was discharged after three weeks of hospitalization. Details of this case are found in reference [3].

**Table 1:** Isolate information and clinical data of necrotizing pneumonia cases.

Case Number	Isolate Name	Country of Origin		Age	Sex	Type of Pneumonia	Culture Origin	MLST	T3SS	Patient Comorbidities	Evidence of Necrotizing Pneumonia	Outcome	Ref.
1	PA1-Riv	France		80	man	CAP	BAL	605	*exoS*	Autoimmune hemolytic anemia	RUL cavitation on CXR	Septic shock requiring vasopressors, discharged from hospital	[3]
2	PA2-Cam	Brazil		44	man	CAP	ETA	377	*exoU*	Tobacco use disorder	RUL lobe parenchymal necrosis and hemorrhagic infiltration on autopsy	Death	[11]
3	PA3-Kot	Japan		29	woman	CAP	Sputum	282	*exoS*	Anorexia nervosa	RUL consolidation and cavitation on CT chest imaging	Death	[12]
4	PA4-Nam	Japan		59	man	CAP	Lung tissue	560	*exoU*	COPD and lung adenocarcinoma	RLL cavitary lung lesions on CT chest imaging	Death	[13]
5	L00-a	USA		52	man	HAP	BAL	532	*exoU*	COVID-19 pneumonia, prolonged intubation, essential hypertension	RLL abscess and hydropneumothoraces on CT imaging	Death	[14]
6	PA6-SCRIPT-94841	USA		45	man	HAP	BAL	242	*exoS*	COVID-19 pneumonia, prolonged intubation	Multiple lung abscesses, bronchopleural fistula and empyema on CT imaging	Death	This study

CAP = community-acquired pneumonia.

HAP = hospital-acquired pneumonia.

BAL = bronchoalveolar lavage.

COPD = chronic obstructive pulmonary disease.

CT = computed tomography.

ETA = endotracheal aspirate.

RLL = right lower lobe.

RUL = right upper lobe.

MLST = multilocus sequence type.

T3SS = type 3 secretion system.

USA= United States of America.

Case 2 A 44-year-old man presented with a complaint of back pain followed by the onset of a dry cough, fever, and hemoptysis. He had a history of smoking and worked in a metallurgic factory. *P. aeruginosa* grew from his blood culture and his tracheal aspirate culture (isolate PA2-Cam)*.* He died 7 h after admission to the hospital. The autopsy revealed pulmonary parenchymal necrosis with hemorrhagic infiltration of the right upper lobe. Details of this case are found in reference [11].

Case 3 A 29-year-old woman presented to the emergency room with a one-day history of general malaise. She had a diagnosis of anorexia nervosa but had no other medical comorbidities. *P. aeruginosa* grew from her blood culture and sputum culture (isolate PA3-Kot). A chest x-ray and chest computed tomography (CT) scan revealed consolidation with a cavity in the right upper lobe. She died four days after hospitalization. Details of this case are found in reference [12].

Case 4 A 59-year-old man presented with a one-week history of shortness of breath. He had a past medical history of chronic obstructive pulmonary disease and recent chemotherapy for lung adenocarcinoma. His CT scan showed cavitary lesions in the right lower lung. He died the following day. *P. aeruginosa* grew from sputum cultures and autopsy cultures of the pulmonary cavitary lesions (isolate PA4-Nam)*.* Details of this case are found in reference [13].

**Fig. 1:**
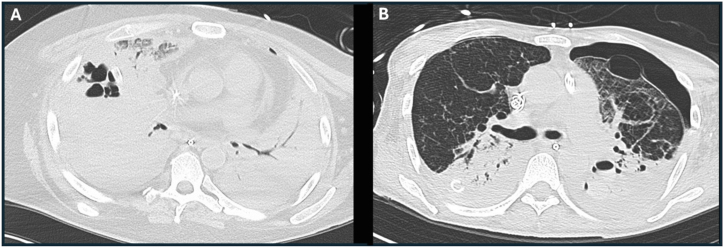
Representative computed tomography (CT) images of two clinical cases of necrotizing pneumonia. **A.** A non-contrast CT chest image from the patient described in case 5. Extensive consolidation of the bilateral lower lobes and multiple cavities in the right lower lobe with air-fluid levels are seen. **B.** A non-contrast CT chest image from the patient described in case 6. The scan is remarkable for bilateral lower lobe consolidation, a chest tube in the right lower lobe, and a hydropneumothorax and cavitary lesions with air-fluid levels in the left lower lobe. The hydropneumothorax was sampled by thoracentesis, and the pleural fluid culture grew *P. aeruginosa*.

Case 5 A 52-year-old previously healthy man was hospitalized with severe COVID-19 pneumonia requiring intubation and ultimately venovenous extracorporeal membrane oxygenation (ECMO). The patient received therapy for SARS-CoV-2 but later developed *P. aeruginosa* pneumonia and bacteremia. CT imaging revealed subsequent development of bilateral lower lobe pneumonia with a right lower lobe abscess and bilateral hydropneumothoraces (Fig. 1A). *P. aeruginosa* grew from the patient's blood culture and BAL culture (isolate L00-a). Care was ultimately withdrawn after the patient suffered an acute brain hemorrhage. Details of this case are found in reference [14].

Case 6 A 45-year-old man with a history of hypertension and renal calculi was hospitalized after 3 days of shortness of breath. He tested positive for SARS-CoV-2 and was treated with remdesivir, dexamethasone, and tocilizumab. The patient had progressively worsening hypoxemia requiring first bilevel positive airway pressure support, then mechanical ventilation two weeks after first being treated for COVID-19 pneumonia. Despite treatment for COVID-19 and *Enterococcus avium* pneumonias, the patient had progressively worsening oxygenation. The patient underwent trials of increased positive end-expiratory pressure, proning, and paralysis. Given the patient's worsening respiratory status, he was transferred on hospital day 23 to Northwestern Memorial Hospital in Chicago, USA. The following day, a bronchoscopy with BAL was performed that yielded fluid negative for SARS-CoV-2 but positive for *P. aeruginosa* by molecular assays. Cefepime had been started a day prior to the bronchoscopy and was continued when the culture of the BAL fluid grew *P. aeruginosa* (20,000 CFU/mL, isolate PA6-SCRIPT-94841) susceptible to this agent. On hospital day 27, the patient was placed on venovenous ECMO. After a lack of clinical improvement, a repeat CT scan of his chest demonstrated lung abscesses, empyema (pleural fluid culture with growth of *P. aeruginosa*), and a bronchopleural fistula (Fig. 1B). On hospital day 30, a repeat BAL was performed because of worsening pressor requirements and leukocytosis. Culture of fluid from the repeat BAL grew *P. aeruginosa* (>100,000 CFU/mL) with intermediate susceptibility to cefepime, and antibiotic therapy was changed to meropenem. Blood cultures were negative throughout the hospital course. The patient developed progressively worsening septic shock, and the family elected to withdraw care. Informed consent was obtained from this patient prior to his death.

### 3.2 Genomic characteristics

**Fig. 2:**
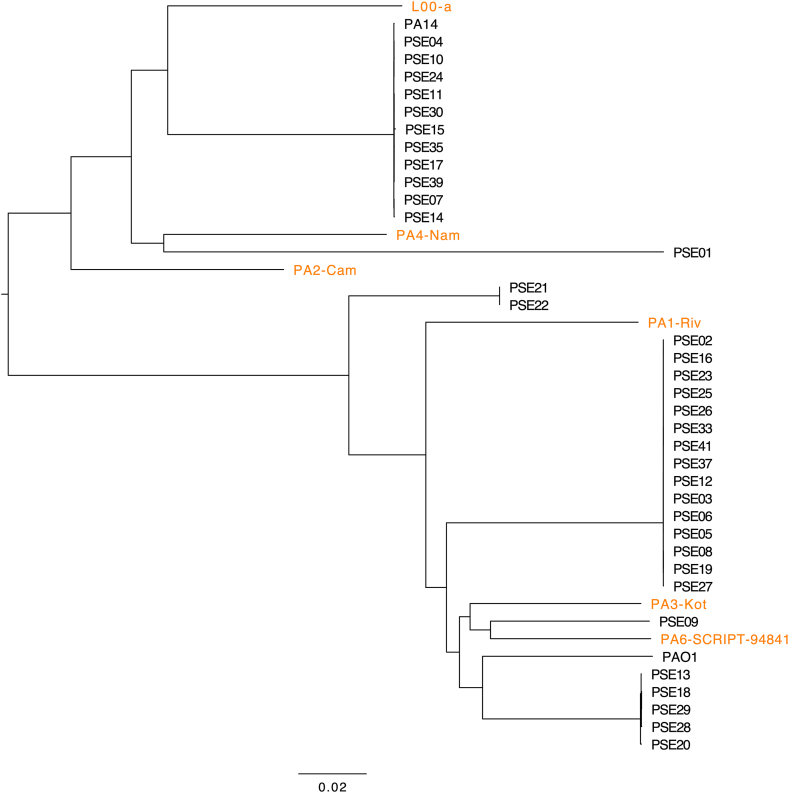
A core-genome unrooted phylogenetic tree of *P. aeruginosa* necrotizing pneumonia (NP) and non-NP isolates. NP isolates (indicated by orange font) were compared to *P. aeruginosa* isolates cultured from the airways of patients with non-necrotizing pneumonia (PSE#) and two commonly used reference strains (PAO1 and PA14). The NP isolates do not share a common lineage.

It is possible that one or several highly virulent lineages of *P. aeruginosa* are responsible for cases of necrotizing pneumonia. For this reason, we sought to examine whether the *P. aeruginosa* strains causing these NP cases were genetically related to each other. For each case, a *P. aeruginosa* isolate from a respiratory specimen (BAL, endotracheal aspirate, sputum, or lung tissue) was obtained (Table 1). We performed short-read genomic sequencing on all the isolates to examine their phylogenetic relationships by generating a tree based on their core genomes (Fig. 2, Supplemental Table 1). For comparison, we included the commonly used reference strains PAO1 and PA14, and thirty-five previously published *P. aeruginosa* respiratory isolates from patients that were not reported as having evidence of necrotizing pneumonia [[15], [16], [17]]. The six NP isolates were not monophyletic but rather distributed across the phylogenic tree. Consistent with this, each isolate had a distinct genotype by MLST (Table 1). From this, we conclude that the NP infections in our study were not caused by a single lineage of *P. aeruginosa.*

We next determined the type-III-secretion genotype of each of the NP isolates. We searched the genomes of these isolates for the *exoU* gene, which encodes for the potent cytotoxin ExoU. Three of the NP isolates (PA2-Cam, PA4-Nam and L00-a) contained the *exoU* gene. The remaining three NP isolates lacked this gene but instead contained the *exoS* effector gene (Table 1). Thus, the presence of the *exoU* gene is not necessary for *P. aeruginosa* strains to cause NP.

### 3.3 Cytotoxicity assays

**Fig. 3:**
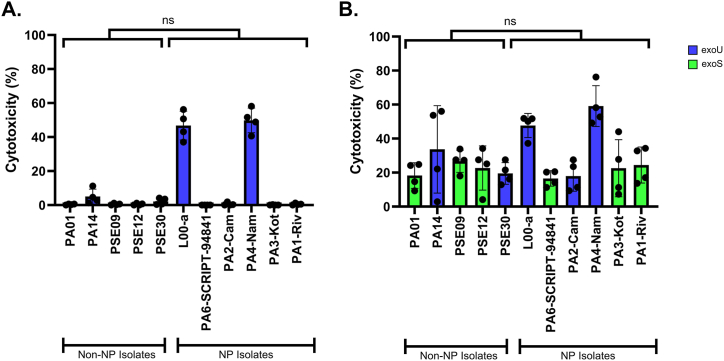
Cytotoxicity of *P. aeruginosa* isolates from patients with and without necrotizing pneumonia (NP). **A.** Three-hour cytotoxicity assays. NP and non-NP isolates were co-incubated with pulmonary epithelial-like A549 cells for 3 hours, and lactate dehydrogenase (LDH) release was measured to quantify the percentage of A549 cells that had lysed. As a group, NP isolates were not statistically more cytotoxic than non-NP isolates. **B.** Eight-hour cytotoxicity assays. As a group, NP isolates were not statistically more cytotoxic than non-NP isolates. *exoU***^*+*^** isolates are shown in blue, and *exoS***^*+*^** isolates are shown in green. Each symbol represents a biological replicate consisting of the mean of four technical replicates. Error bars represent standard errors of the mean. ns = not significant.

We next examined whether the NP isolates shared the capacity to cause rapid lysis of mammalian cells, a property that might explain their association with necrotizing pneumonia. Each isolate was incubated with A549 pulmonary epithelial-like cells for 3 or 8 hours, and cell lysis was measured. At the 3-h timepoint, the NP isolates as a group showed a trend towards a higher mean percent cytotoxicity than the non-NP isolates, although this difference was not statistically significant (16.3 % versus 1.7 %, p = 0.23, Fig. 3A, Supplemental Table 2). This difference was driven by two *exoU* ^*+*^ NP isolates, PA4-Nam and L00-a, each of which caused relatively high levels of cell lysis, whereas the remaining NP isolates caused only minimal cytotoxicity. In contrast, all the non-NP isolates caused only minimal cytotoxicity, including the *exoU*^*+*^ isolates PSE30 and PA14. Thus, the non-statistically significant difference in aggregate cytotoxicity between the NP and non-NP isolates was primarily driven by the cytotoxicity difference between the NP *exoU*^*+*^ and non-NP *exoU*^*+*^ isolates (32.4% versus 3.6%; p = 0.26). By the 8-h timepoint, the *exoU***^*-*^** isolates had begun to cause cell lysis, and the cytotoxicity difference between the NP and non-NP isolates was less (31.4 % vs. 25.2 %, p = 0.51; Fig. 3B), including between the *exoU*^*+*^ NP isolates and the *exoU*^*+*^ non-NP isolates (41.6% vs. 26.6%, p = 0.43). These findings indicate that not all necrotizing pneumonia isolates are capable of rapidly lysing epithelial-like cells *in vitro.* However, some *exoU* ^*+*^ NP isolates are highly cytotoxic.

### 3.4 Virulence in a *G. mellonella* model of infection

**Fig. 4:**
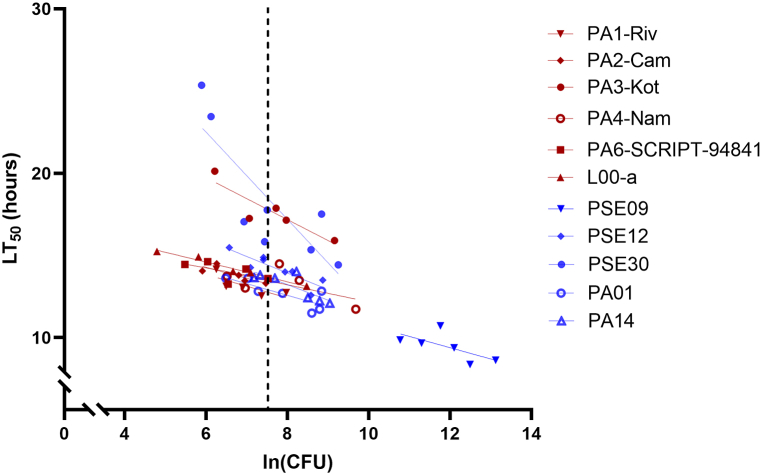
Virulence of *P. aeruginosa* strains in *Galleria mellonella* larvae. Larvae were injected with varying doses of each *P. aeruginosa* isolate, and the 50% lethal time (LT_50_) was measured. Linear regression was used to fit lines to the data points. The LT_50_ value at a dose of 2000 colony-forming units (CFU) was then calculated to compare the virulence of the isolates. Each symbol represents the LT_50_ of 9–10 larvae injected with a single dose of the indicated strain of *P. aeruginosa.* No difference in mean LT_50_ values between NP isolates (shown in brown) and non-NP isolates (shown in blue) was observed (14.0 hr vs. 14.0 hr, respectively).

To better assess the overall virulence of the *P. aeruginosa* isolates, we tested them in *G*. *mellonella* larvae. This invertebrate model yields estimates of *P. aeruginosa* virulence that correlate with those obtained in mice [26]. Because *G. mellonella* is much more susceptible to *P. aeruginosa* than mice, we measured the time required for 50% of larvae to die (50% lethal time; LT_50_) following injection of different numbers of *P. aeruginosa* bacteria rather than a 50% lethal dose [25]. At a given dose, more virulent strains of *P. aeruginosa* kill *G. mellonella* more rapidly. We compared the virulence of the NP strains to those of the non-NP strains PAO1, PA14, PSE09, PSE12, and PSE30. At an extrapolated dose of 2000 CFU, the mean LT_50_ was the same for the NP isolates and the control isolates (14.0 hr; Fig. 4, Supplemental Table 3). One NP isolate (PA3-Kot) and one non-NP isolate (PSE30) were outliers, with substantially longer LT_50_ values (lower virulence) than the other NP isolates. These findings suggest that NP isolates are not more virulent than other *P. aeruginosa* isolates in the *G. mellonella* model system.

## 4 Discussion

NP caused by *P. aeruginosa* is relatively rare, but we were able to obtain isolates from 6 globally distributed cases, 5 of which were previously reported in the literature, and one new case from our institution. We examined these isolates to determine whether they exhibited unique genotypic or pathogenic characteristics. Notably, no phylogenetic relationships between these clinical isolates were observed, and each belonged to a distinct sequence type. The vast majority of *P. aeruginosa* strains fall into one of two large, evolutionarily divergent genetic clades distinguished by the presence of the *exoU* gene or the *exoS* gene [27], both of which encode effector proteins of the type III secretion system. The NP isolates were not relegated to one or the other of these two large clades but rather were found in both. This implies that a single highly virulent clone of *P. aeruginosa* is not responsible for all NP cases and that both *exoU*^*+*^ and *exoS*^*+*^ strains are capable of causing NP.

Another possibility is that NP isolates, although phylogenetically distinct, share the common trait of being highly aggressive in their ability to infect the host. Such a trait could have developed through convergent evolution or acquisition of accessory genes via horizontal gene transfer. To test whether NP isolates were indeed more virulent than other isolates, we used a *G. mellonella* larva model of infection. This model has been used extensively to study bacterial virulence [28]. However, the NP isolates as a group did not differ from non-NP clinical isolates or laboratory strains in this model. This indicates that at least some NP isolates do not have highly virulent phenotypes in the context of the *G. mellonella* model.

The *P. aeruginosa* isolates (including the NP isolates) varied in their ability to lyse mammalian cells under cell culture conditions, but as a group NP isolates were not more cytotoxic than non-NP isolates (Fig. 3). As expected, those isolates containing the gene encoding the potent cytotoxin ExoU tended to cause more cell lysis than those that lacked this gene. Similar results have been reported with other isolates and cell lines [17,29,30]. However, among *exoU* ^+^ isolates, two NP isolates were particularly cytotoxic at earlier timepoints. Although the number of strains available for testing in this exploratory study was too small to draw conclusions, it is possible that a subset of NP isolates have the capacity to rapidly lyse pulmonary cells and thus cause the formation of necrotic lesions in the lungs. Larger studies using additional *exoU*^*+*^ NP and non-NP strains are necessary to test this hypothesis. Alternatively, *in vitro* cytotoxicity may not be an appropriate biomarker for the tissue destruction observed in NP. Assays measuring resistance to phagocytosis, serum sensitivity, and biofilm formation may be more informative. Similarly, virulence factors other than ExoU and ExoS, such as exotoxin A, elastase, pyoverdine, pyocyanin, phospholipases, or alkaline protease, may be responsible for the severity of these infections.

In summary*,* we did not detect a particular *P. aeruginosa* characteristic such as enhanced virulence, cytotoxicity, or phylogenetic clustering that was associated with NP pathology. Larger studies are necessary to better define the role of bacterial cytotoxicity in NP.

## CRediT authorship contribution statement

**T.C. Bolig:** Writing – review & editing, Writing – original draft, Methodology, Investigation, Formal analysis, Data curation. **S.H. Nozick:** Writing – review & editing, Investigation. **C.M.R. Axline:** Investigation. **I. Niki:** Investigation. **A. Valdes:** Investigation. **A. Alisoltanidehkordi:** Software, Formal analysis. **T.L. Turner:** Investigation. **F.P.F. Campos:** Resources. **H. Namkoong:** Resources. **P. Riviere:** Resources. **J. Rello:** Resources. **E. Ozer:** Validation, Software, Formal analysis. **R. Wunderink:** Writing – review & editing, Supervision, Resources, Project administration, Methodology, Funding acquisition, Data curation, Conceptualization. **A.R. Hauser:** Investigation.

## Funding STATEMENT

Support for this work was provided by the 10.13039/100000002National Institutes of Health awards RO1 AI118257, K24 AI04831, R21 AI129167 and R21 AI153953 (all to 10.13039/501100001036ARH) and U19 AI135964 (RGW and 10.13039/501100001036ARH).

## Declaration of competing interest

The authors declare that they have no known competing financial interests or personal relationships that could have appeared to influence the work reported in this paper.

## Data Availability

The data that support the findings of this study are openly available in PRISM at https://prism.northwestern.edu, reference number https://doi.org/10.18131/wy6v0-hqk52.
