# Supplementary material for: Cytotoxicity and virulence attributes of Pseudomonas aeruginosa isolates from case reports of patients with necrotizing pneumonia
Source: Respir Med Case Rep. 2025 Nov 19;58:102329. doi: 10.1016/j.rmcr.2025.102329 (PMC12702255; doi:10.1016/j.rmcr.2025.102329)
Supplement: Multimedia component 1 [file mmc1.docx]

**Supplemental Table 1. Sequencing characteristics of *P. aeruginosa* isolates**

| **Supplemental Table 2A. Three-hour cytotoxicity data for individual experiments** | | | | | |
| --- | --- | --- | --- | --- | --- |
| **Strain** | **Percent Cytotoxicity** | |  |  |  |
|  | Experiment 1* | Experiment 2 | Experiment 3 | Experiment 4 | Mean Percent Cytotoxicity |
| PA01 | 0 | 0.6 | 0.3 | 0.6 | 0.4 |
| PA14 | 1.3 | 4.6 | 2.9 | 11.2 | 5 |
| PSE09 | 0.3 | 0 | 0.6 | 1.1 | 0.5 |
| PSE12 | 1.1 | 0 | 0.7 | 0 | 0.5 |
| PSE30 | 3.5 | 0.6 | 4.1 | 0.4 | 2.2 |
| L00-a | 50.7 | 56.2 | 43.1 | 37 | 46.8 |
| PA6-SCRIPT-94841 | 0 | 0 | 0 | 0 | 0 |
| PA2-Cam | 0.3 | 1.9 | 0 | 0 | 0.6 |
| PA4-Nam | 58.1 | 48.9 | 51.6 | 40.6 | 49.8 |
| PA3-Kot | 0 | 0.3 | 0 | 0 | 0.1 |
| PA1-Riv | 0.5 | 1.2 | 0 | 0.4 | 0.5 |
| **Supplemental Table 2B. Eight-hour cytotoxicity data for individual experiments** | | | | | |
| **Strain** | **Percent Cytotoxicity** | |  |  |  |
|  | Experiment 1* | Experiment 2 | Experiment 3 | Experiment 4 | Mean Percent Cytotoxicity |
| PA01 | 9.3 | 24.8 | 24.2 | 14.7 | 18.3 |
| PA14 | 56 | 22.1 | 2.9 | 53.7 | 33.7 |
| PSE09 | 27.5 | 18 | 27.3 | 33.9 | 26.7 |
| PSE12 | 25.1 | 4.1 | 34.2 | 27.5 | 22.7 |
| PSE30 | 21.5 | 16.2 | 27.5 | 12.9 | 19.5 |
| L00-a | 50.1 | 37.2 | 51.7 | 51.9 | 47.7 |
| PA6-SCRIPT-94841 | 14.2 | 20.5 | 20.3 | 11.3 | 16.6 |
| PA2-Cam | 23.5 | 27.5 | 9.4 | 11.4 | 18.0 |
| PA4-Nam | 52.8 | 76.2 | 49.2 | 58.2 | 59.1 |
| PA3-Kot | 27.8 | 44 | 11.3 | 7.3 | 22.6 |
| PA1-Riv | 34.3 | 32.8 | 17 | 13.7 | 24.5 |

*The mean cytotoxicity for each strain was based on four biological replicates.

**Supplemental Table 3. Data used to calculate *G. mellonella* larvae LT_50_ values**

| **Strain** | **Dose (CFU)** | **ln(Dose)** | **LT_50_ (hr)** | **Regression line** | | | **Calculated LT_50_ at dose of 2000 CFU (hr)** |
| --- | --- | --- | --- | --- | --- | --- | --- |
|  |  |  |  | **slope** | | **y-intercept** |  |
| PA3-Kot | 9500 | 9.16 | 15.90 | -1.27 | 27.36 | | 17.69 |
|  | 2250 | 7.72 | 17.87 |  |  | |  |
|  | 1160 | 7.06 | 17.25 |  |  | |  |
|  | 2900 | 7.97 | 17.14 |  |  | |  |
|  | 500 | 6.21 | 20.13 |  |  | |  |
| PA6-SCRIPT-94841 | 1850 | 7.52 | 13.59 | -0.44 | 16.90 | | 13.53 |
|  | 695 | 6.54 | 13.26 |  |  | |  |
|  | 1070 | 6.98 | 14.17 |  |  | |  |
|  | 420 | 6.04 | 14.61 |  |  | |  |
|  | 240 | 5.48 | 14.45 |  |  | |  |
| L00-a | 4750 | 8.47 | 13.12 | -0.60 | 18.19 | | 13.63 |
|  | 1200 | 7.09 | 13.96 |  |  | |  |
|  | 780 | 6.66 | 14.03 |  |  | |  |
|  | 335 | 5.81 | 14.91 |  |  | |  |
|  | 120 | 4.79 | 15.24 |  |  | |  |
| PA1-Riv | 1565 | 7.36 | 12.54 | -0.71 | 18.08 | | 12.69 |
|  | 990 | 6.90 | 13.07 |  |  | |  |
|  | 520 | 6.25 | 14.16 |  |  | |  |
|  | 2900 | 7.97 | 12.74 |  |  | |  |
|  | 660 | 6.49 | 13.12 |  |  | |  |
| PA2-Cam | 1750 | 7.47 | 13.30 | -0.66 | 18.24 | | 13.21 |
|  | 895 | 6.80 | 13.79 |  |  | |  |
|  | 525 | 6.26 | 14.50 |  |  | |  |
|  | 1040 | 6.95 | 13.46 |  |  | |  |
|  | 370 | 5.91 | 14.06 |  |  | |  |
| PSE12 | 5350 | 8.58 | 12.58 | -1.00 | 18.48 | | 10.84 |
|  | 3350 | 8.12 | 14.02 |  |  | |  |
|  | 1200 | 7.09 | 14.26 |  |  | |  |
|  | 710 | 6.57 | 15.48 |  |  | |  |
|  | 7150 | 8.87 | 13.49 |  |  | |  |
|  | 2800 | 7.94 | 14.00 |  |  | |  |
|  | 1650 | 7.41 | 14.88 |  |  | |  |
|  | 1650 | 7.41 | 14.73 |  |  | |  |
| PSE30 | 6900 | 8.84 | 17.51 | -2.65 | 38.40 | | 18.25 |
|  | 1800 | 7.50 | 17.77 |  |  | |  |
|  | 455 | 6.12 | 23.44 |  |  | |  |
|  | 360 | 5.89 | 25.35 |  |  | |  |
|  | 10400 | 9.25 | 14.42 |  |  | |  |
|  | 5350 | 8.58 | 15.34 |  |  | |  |
|  | 1695 | 7.44 | 15.83 |  |  | |  |
|  | 1025 | 6.93 | 17.05 |  |  | |  |
| PSE09 | 265000 | 12.49 | 8.36 | -0.69 | 17.64 | | 12.41 |
|  | 80500 | 11.30 | 9.67 |  |  | |  |
|  | 47500 | 10.77 | 9.86 |  |  | |  |
|  | 500000 | 13.12 | 8.63 |  |  | |  |
|  | 180000 | 12.10 | 9.38 |  |  | |  |
|  | 127500 | 11.76 | 10.71 |  |  | |  |
| PA01 | 6600 | 8.79 | 11.73 | -0.71 | 18.23 | | 12.85 |
|  | 6900 | 8.84 | 12.82 |  |  | |  |
|  | 1455 | 7.28 | 12.81 |  |  | |  |
|  | 5450 | 8.60 | 11.49 |  |  | |  |
|  | 2650 | 7.88 | 12.70 |  |  | |  |
|  | 1125 | 7.03 | 13.61 |  |  | |  |
|  | 650 | 6.48 | 13.61 |  |  | |  |
| PA14 | 6600 | 8.79 | 12.23 | -0.95 | 20.82 | | 13.60 |
|  | 3700 | 8.22 | 14.02 |  |  | |  |
|  | 1525 | 7.33 | 13.81 |  |  | |  |
|  | 8400 | 9.04 | 12.09 |  |  | |  |
|  | 4900 | 8.50 | 12.42 |  |  | |  |
|  | 2190 | 7.69 | 13.61 |  |  | |  |
|  | 1315 | 7.18 | 13.65 |  |  | |  |
| PA4-Nam | 16000 | 9.68 | 11.73 | -0.52 | 17.34 | | 13.42 |
|  | 4000 | 8.29 | 13.48 |  |  | |  |
|  | 2450 | 7.80 | 14.48 |  |  | |  |
|  | 1055 | 6.96 | 13.02 |  |  | |  |
|  | 670 | 6.51 | 13.73 |  |  | |  |
